# Metallothionein and Cadmium Toxicology—Historical Review and Commentary

**DOI:** 10.3390/biom12030360

**Published:** 2022-02-24

**Authors:** Monica Nordberg, Gunnar F. Nordberg

**Affiliations:** 1 Institute of Environmental Medicine, Karolinska Institutet, SE-17177 Stockholm, Sweden; 2 Division of Sustainable Health, Department of Public Health and Clinical Medicine, Umeå University, SE-90187 Umeå, Sweden; gunnar.nordberg@umu.se

**Keywords:** cadmium toxicity, metallothionein, cadmium and zinc in metallothionein, cadmium binding in blood plasma, toxicokinetic model for cadmium, kidney toxicity of cadmium, metallothionein gene expression in lymphocytes, metallothionein autoantibodies, cadmium risk assessment

## Abstract

More than one and a half centuries ago, adverse human health effects were reported after use of a cadmium-containing silver polishing agent. Long-term cadmium exposure gives rise to kidney or bone disease, reproductive toxicity and cancer in animals and humans. At present, high human exposures to cadmium occur in small-scale mining, underlining the need for preventive measures. This is particularly urgent in view of the growing demand for minerals and metals in global climate change mitigation. This review deals with a specific part of cadmium toxicology that is important for understanding when toxic effects appear and, thus, is crucial for risk assessment. The discovery of the low-molecular-weight protein metallothionein (MT) in 1957 was an important milestone because, when this protein binds cadmium, it modifies cellular cadmium toxicity. The present authors contributed evidence in the 1970s concerning cadmium binding to MT and synthesis of the protein in tissues. We showed that binding of cadmium to metallothionein in tissues prevented some toxic effects, but that metallothionein can increase the transport of cadmium to the kidneys. Special studies showed the importance of the Cd/Zn ratio in MT for expression of toxicity in the kidneys. We also developed models of cadmium toxicokinetics based on our MT-related findings. This model combined with estimates of tissue levels giving rise to toxicity, made it possible to calculate expected risks in relation to exposure. Other scientists developed these models further and international organizations have successfully used these amended models in recent publications. Our contributions in recent decades included studies in humans of MT-related biomarkers showing the importance of MT gene expression in lymphocytes and MT autoantibodies for risks of Cd-related adverse effects in cadmium-exposed population groups. In a study of the impact of zinc status on the risk of kidney dysfunction in a cadmium-exposed group, the risks were low when zinc status was good and high when zinc status was poor. The present review summarizes this evidence in a risk assessment context and calls for its application in order to improve preventive measures against adverse effects of cadmium exposures in humans and animals.

## 1. Introduction

Cadmium (Cd) is a toxic metal and adverse human health effects have been known for more than one and a half centuries [1]. Governments and responsible authorities in many countries made considerable efforts in controlling exposures and prevent adverse health effects. However, in some countries there is artisanal and small-scale mining (ASM) where uncontrolled exposures to cadmium and many other metals occur [2]. There is an urgent need for adequate risk assessments and preventive measures in ASM, particularly in the context of growing demand for minerals and metals for global climate change mitigation. The present review focuses on a specific part of cadmium toxicology that is important for understanding of how toxic effects occur and how serious they will be at various exposure levels. Such information is crucial for risk assessment. In addition to the acute gastrointestinal and respiratory effects reported by clinical doctors in 1858 [1], the toxic effects of cadmium in exposed animals and humans include lung, kidney and bone disease, reproductive toxicity and cancer [3]. Since 1957 [4], increasing evidence has been accumulating on the role of the Metallothioneins (MTs) in cadmium toxicology. MTs are low-molecular-weight cadmium-binding proteins occurring in human and animal tissues. Piscator 1964 [5] suggested that binding of Cd to MTs modifies the toxicity of cadmium. The present authors contributed evidence during the first two decades after the discovery of MTs, concerning Cd binding to MTs in tissues in relation to Cd exposure [6,7]. In addition, we described the role of MT in Cd transport and uptake in the kidney [8,9] and their likely role in modulating the interaction of Cd with intracellular targets of importance for expression of toxicity. We have continuously contributed to the knowledge about cadmium toxicology also in the last four decades and the present review and commentary summarizes our findings and gives our views on the role of metallothionein in cadmium toxicology as applied to risk assessment. Other reviews give detailed chemical properties of metallothionein [10] and detailed molecular pathways of importance for Cd kinetics and toxicity [11], not yet fully used in risk assessment.

## 2. Metallothioneins, Their Discovery, Isolation and Chemical Properties

In 1957 Margoshes and Vallee [1] published data on a Cd binding protein in equine renal tissue, containing a high natural content of Cd and zinc (Zn). In 1960 [12] and 1961, Kägi and Vallee [13] published the first detailed characterization of the protein from horse kidneys and named it Metallothionein (MT). In 1964, Piscator [5] described that MT could be induced by Cd exposure in rabbits, and in 1972, Nordberg et al. [7] isolated three forms of MT by isoelectric focusing. The pI of these three forms were 3.9, 4.5 and 6.0, respectively. We characterized the two main forms by amino acid analysis. This and later research demonstrated that MTs are low molecular weight (about 6500 Da varying depending on the metal content), cysteine-rich metal-binding proteins. A wide variety of organisms contain these proteins, including bacteria, fungi and all eukaryotes, i.e., plant and animal species [10,14].

MTs are of importance for toxicokinetics and biochemistry of essential and non-essential metals. Metallic species of Zn, Cd, mercury and copper bind to MT in clusters (see below) Other metals/metalloids such as selenium and bismuth are also bound to MT in vivo, but the exact nature of such binding has not been characterized in detail. Although they are mainly intracellular proteins, MTs have been detected in small amounts in blood and urine. MTs are determined in blood and tissues by biochemical and immunological methods [15].

Four forms of MTs, i.e., MT 1–4 have been identified. MT-1 and MT-2 are the most studied forms, expressed in most tissues and they both consist of 61 aminoacids (aa). Several isoforms of MT-1 have been identified. MT-3 occurs in brain tissue, has 68 aa and is rich in zinc. It is sometimes called Growth Inhibitor Factor, GIF. MT-4 is expressed in keratinocytes and has 64 aa. MT-1 and MT-2 have 20 cysteine residues (30%), they contain N-acetylmethionine and C-alanine but no aromatics, no histidine. The aminoacid sequence is unique and the tertiary structure displays metal clusters. MT-1 and MT-2 have two clusters A and B with four and three metals, respectively. The C-terminal is part of the A cluster and the N-terminal of the protein forms the B-cluster [16]. Zn, Cd, Hg, Cu makes up 5–10% *w*/*w*. UV absorption varies depending on metal bound, it is (in nm) 225 for Zn-MT, 250 for Cd-MT, 300 for Hg-MT and 275 for Cu-MT [14,17].

The link between MT and DNA for MT-1, -2, -3, -4 is related to age; fetus, newborn, adult. Gender aspects, i.e., differences exist between men and women. MT levels are higher in liver tissue of women than in men. In iron deficiency there is increased MT-1 in bone marrow and in kidney MT is decreased. There is genetic polymorphism, with several genes for MT located on same chromosome. It is possible that they are coding for specific MT-functions [14,17].

MT is present in liver, kidney, urine, plasma and blood. It serves in several functions including transport of metals e. g. Cd, Cu, Zn. Another role is in detoxification of metals e.g., Cd, Zn and Hg. Non-MT-bound metal species are more toxic than MT-bound metal, the latter form accumulates in tissues. MT also serves as a free radical scavenger, it serves in storage of metals, metabolism of essential metals and has functions related to the immune response. Metal binding to MT modifies genotoxicity and carcinogenicity [14,17].

The present review summarizes experimental evidence and observations in humans concerning protein binding of Cd in blood and tissues. Because the kidney has been considered the critical organ in long-term cadmium exposures, particular attention is given to concentrations of Cd, Zn and MT in kidneys and the appearance of tubular proteinuria. Data reviewed in the following sections are from studies performed during 50 years. All studies on animals and humans had permissions from the appropriate ethical committees.

Development in MT research since 1970′s focused on purification, identification and nomenclature, characterization, molecular biology, confirmation of results in toxicology and chemical/biochemical characterization, discussed during the first international meeting on metallothionein in 1978 [18]. Outcome of this workshop was to conclude on a terminology of the protein metallothionein. 

Purification and identification of metallothionein in biological tissues caused in the beginning problems. In the 1970s, homogenization, ultrafiltration and gelchromatography were the conventional ways of purification. It was found that storage of the 105,000 g of supernatant at various time and temperature influenced greatly the outcome of protein separation which is important to be aware of also today. Recording of the absorbance at 250 and 280 nm, the ratio indicating the purity of MT and was monitored during gelchromatography. It was found that when storage had been in room temperature, the Cd-MT peak apeared at higher molecular weight than where the MT was normally eluted, when samples were kept refrigerated (+5 °C). Gelchromatography has to be performed at such temperature. However, by adding mercaptoethanol to the supernatant, polymerization was reversed and the MT peak was at its normal elution volume. Our preliminary attempts to study MT by polyacrylamide gel electrophoresis were unsuccessful because of the difficulties to avoid oxidation. Storage in low-temperature freezers was useful. After a procedure of freezing the supernatants from tissue homogenates dropwise in liquid nitrogen and storage at minus 65 degrees Celsius, the distribution pattern of absorbance ratio and of distribution pattern of cadmium was shown to be unchanged and appeared as the same for samples taken directly for protein separation. It turned out to be very effective and useful in studying Cd and MT in tissue samples with a low concentration of both. Some studies used radiolabeling of MT with Cd^109^, which is excellent to study low concentrations of Cd in biological tissues [8]. Radiolabelling with Cd showed that of the seven metals one zinc always had to be part of the protein. It also explained the success in the studies of binding of Cd to MT and the kinetics of MT in blood and in plasma. When these procedures were not used, misinterpretations and misleading data have been reported in the literature. Freeze-dried MT can be stored in hermetic vials at −80 °C for a very long time without oxidation of the protein.

## 3. Cadmium Toxicokinetics—Role of Metallothioneins

### 3.1. Cd Uptake

Uptake of Cd from the skin into the blood is limited after dermal application. Inhalation is the main route of uptake after exposure to airborne particulate Cd in industrial environments and is also an important route for tobacco smokers. Between 7 and 40 percent of inhaled cadmium will be taken up into blood; the higher percentages are valid for soluble cadmium compounds and nanopariculate cadmium, for example, in cigarette smoke [19]. Cd binds to MT in lung tissue and MT is induced by Cd exposure [20]. Binding to MT modifies the toxic effects in pulmonary tissues.

Studies in humans of the uptake of Cd from the gastrointestinal tract into the systemic circulation showed approximately 5 percent uptake for men and 10 percent for women. Young women with low iron stores may take up as much as 40 percent of dietary cadmium (review [3]). There are data in animals showing a similar percentage of systemic uptake of MT-bound Cd as for other chemical species of cadmium when introduced into the gastrointestinal tract, but the systemic distribution is different (see Section 3.2.) and part of ingested CdMT is taken up intact into blood. Increased uptake of non-MT cadmium from the diet occurs in animals when intake of iron, zinc, calcium or protein is low (review [3]). Experimental studies reported that a number of pathways for essential metals such as DMT 1 [21,22], CaT1 [23] and ZIP8 and ZIP14 [24] are involved in Cd uptake. Ohta and Ohba 2020 [25] cited a number of authors who had reported involvement of additional pathways in intestinal cadmium uptake (ZIP4, ZnT1, ATP7A; TRVP6) and they performed in vivo studies in animals with increasing oral Cd^2+^ doses and found related increased Cd concentrations and increased gene expression of MT-1, MT-2 and ZIP14, DMT1, ATP7A and TRVP6, particularly in the duodenal tissue. The exact role of these proteins/transporters for Cd uptake still is not fully clarified.

### 3.2. Cd in Blood and Transport to Tissues

Adverse effects of Cd occur to a large extent after systemic distribution to various tissues such as the kidneys, the skeleton and other organs. Transfer through blood is a major route of distribution. The low concentrations in plasma in combination with insufficient sensitivity of chemical analytical methods made it difficult for a long time to perform adequate studies of the chemical concentration and the binding of Cd in blood plasma. Friberg 1952 [26] showed a long time ago that Cd is mainly found in red blood cells in experimental animals. The use of radiolabeled Cd in combination with gel chromatography offered an opportunity also to study the binding to proteins in plasma [8,27,28].

**Figure 1:**
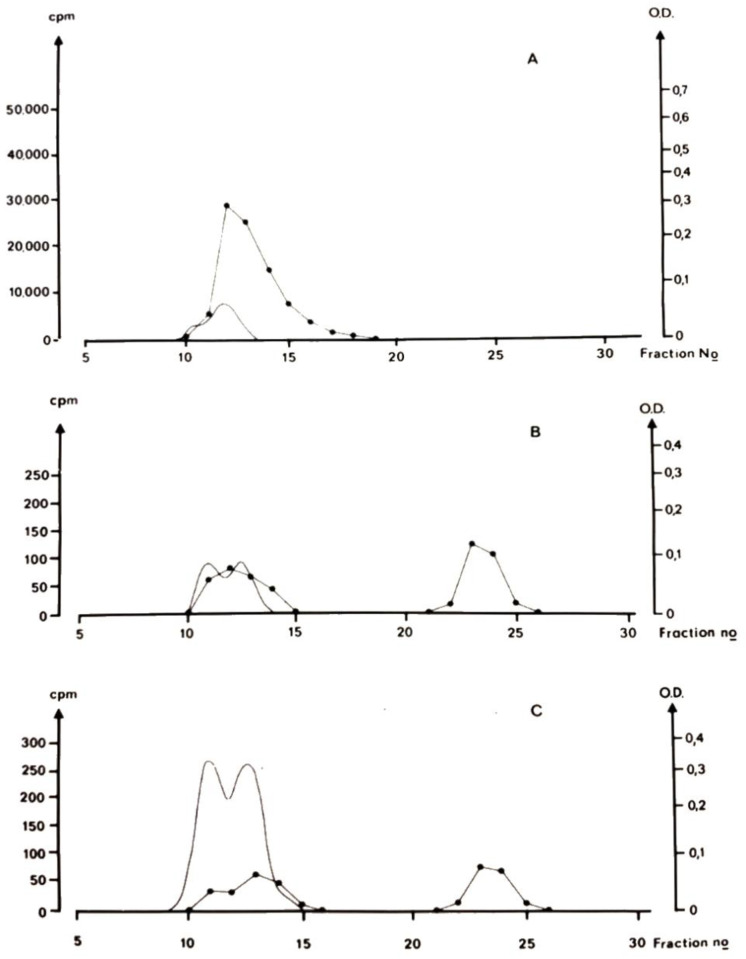
Binding of cadmium in blood plasma. The panels show the results of gel-chromatographic (G75) separation (at +5 °C) of blood plasma from mice at various time points after s.c. injection of a single dose of radiolabeled CdCl_2_. (**A**): 20 min after injection, (**B**): 96 h after injection, (**C**): 192 h after injection. At the shorter time (20 min) all Cd appeared in a high molecularweight peak (fractions 12–14). At longer longer times (**B**,**C**), when the concentration of Cd in plasma was 9 nanomol/kg, a considerable proportion of plasma Cd was detected in a second peak (fractions 23–24) at the molecular size of MT. Line with dots: radiocadmium, unbroken line optical density 254 nm (OD). (Picture of Original drawing of chromatographic results. Experimental details described in [27]).

Figure 1 shows that after a single dose of ionic Cd, binding is initially predominantly to high molecular mass proteins, probably albumin and at longer time intervals (96 and 192 h) after administration, a considerable proportion of plasma cadmium occurs at a molecular size of MT [24]. The occurrence of Cd bound to a protein of the size of metallothionein indicates an important role for this binding form for transport of Cd to the kidney. Like other very small proteins, MT passes through the kidney glomerular membrane into primary urine. CdMT is subsequently reabsorbed into proximal tubular cells. CdMT transport from the blood to renal tubular cells is rapid and almost complete [8,29]. Other species of Cd, for example, Cd-albumin in blood plasma does not enter the kidneys to the same extent. An example is the different kidney accumulation of Cd in animals fed CdMT and other animals fed cadmium chloride [30]. Part of the CdMT enters blood in this form, which is accumulated in the renal cortex while Cd from CdCl_2_ binds to albumin in blood and is accumulated mainly in the liver [27]. After a single administration of Cd^2+^, there is a redistribution of Cd from the liver to the kidney with time (see subsequent paragraph). This redistribution is related to the time-dependent change in binding in blood plasma (Figure 1).

As mentioned, the concentration of Cd in blood cells is considerably higher than it is in plasma. In the experiments described in Figure 1, blood cell Cd was 100 times higher than plasma concentrations at 96 h and longer. The binding of Cd in blood cells was also studied. A major part of Cd was bound to a protein with the same molecular size as MT, and not mainly to the fractions where hemoglobin was eluted [27]. Although Cd binding to the small protein in blood cells with the same size as MT, does not have an immediate impact on renal Cd accumulation, the gradual breakdown of blood cells will mean a slow release that will possibly also end up in the proximal tubules of the kidney. A role for CdMT in transport of Cd to the kidney is presently widely accepted as a probable course of events also in humans [11], but as pointed out by Thévenod and Wolff [11], chromatographic evidence in humans is lacking. On the other hand, MT has been detected by imunologic methods in human blood sera from normal and occupationally Cd-exposed humans [31,32] and it seems likely that it would bind Cd. CdMT occurs in human urine [31] (see also Section 3.3). As mentioned in the introduction to this section, because of the limited sensitivity of methods for chemical analysis of Cd, it has not been possible to study cadmium binding to plasma proteins in humans at existing exposure levels. Recently, Li et al., 2021 [33] reported that in 11 out of 29 blood samples (average plasma Cd 0.08 ng/mL) cadmium appeared to be bound to Apo-lipoprotein A1 (ApoA1). They were unable to identify Cd-binding proteins in crude plasma samples and used procedures to remove major proteins from plasma before examining the remaining proteins. It is unclear whether these authors took precautions to avoid oxidation and polymerization of MT and it is possible that the procedures to remove major proteins influenced Cd distribution among proteins. It would be interesting to examine this possibility in future studies. 

### 3.3. Cadmium Distribution among Organs

After a single exposure to inorganic Cd salts in experimental animals, there is initially a high Cd concentration in the liver, decreasing with time. A redistribution occurs to the kidney and this organ later displays the highest concentration among body organs [34,35,36]. The increase in the kidney Cd concentration can continue for months after a single exposure. Organ distribution is dose dependent. After high doses, regardless of exposure route, there is a larger proportion of Cd in the liver than at lower doses. At low doses, the accumulation in the kidney is more prominent, e.g., [37] Additionally, in long-term exposure, the kidney has the highest concentration of Cd [36]. Piscator 1964 [5] and Nordberg et al. [6] examined the binding of Cd in liver tissue of Cd exposed experimental animals and found a major proportion of Cd bound to MT. Repeated exposure to cadmium gave rise to higher levels of Cd and MT in the liver thus demonstrating that Cd exposure induced the synthesis of MT in that tissue. The authors considered that the binding of cadmium to MT is of considerable importance for cadmium toxicology. Cd exposure induces the synthesis of MT-1 and MT-2 in many tissues in animals and humans (Section 2). As mentioned (Figure 1) a proportion of blood Cd, both in hemolysate from blood cells and in blood plasma, is bound to a MT-like protein. Thus, a likely explanation for the redistribution of Cd from liver to kidney is a release of CdMT from the liver and transport to the kidney by glomerular filtration and reabsorption in the kidney tubules. Transport of injected CdMT, isolated from Cd-exposed animals, was quick from blood into the kidney. Approximately 95 percent of the injected dose is taken up by renal tubules [9,29]. Uptake into proximal tubular cells occurs through the megalin: cubilin-receptor-mediated endocytosis (review [11,38]). Cd build-up in these cells stimulates MT-synthesis, and a continuous rebinding to MT takes place in these cells. This explains why the biological half-life of Cd in such cells is so long. In humans, the half-life is estimated to be 10–30 years. Thus, at background exposures, Cd accumulates continuously during the human life-span. When the concentration of Cd in the kidney cortex increases, a critical concentration is reached, and kidney dysfunction appears (see Section 4.1). 

**Figure 2:**
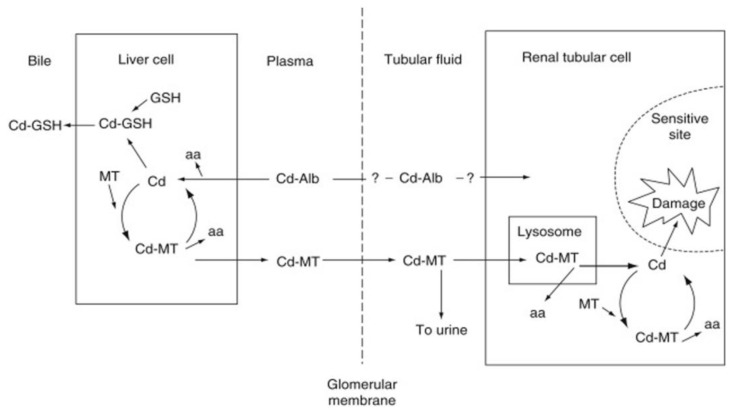
Basic flow scheme of Cd in the body demonstrating the role of binding forms in blood and MT synthesis and degradation. aa, amino acids; Alb, albumin GSH, glutathione; MT, metallothionein. Modified from [39].

Figure 2 describes a scheme of the likely flow of albumin-bound Cd from plasma to the liver, where Cd-albumin is taken up and degraded, the released Cd^2+^ induces the synthesis of MT and binds to newly synthesized MT. 

Thus, in continuous exposure, CdMT is the dominating form of Cd in the liver. Subsequently a small proportion of liver CdMT enters plasma from where it is filtered through the glomerular membrane and taken up in kidney tubules where cellular damage may ensue (Section 4.1). First presented in 1984 by one of the present authors [39], this scheme has been widely accepted and supported by data contributed by other scientists.

Chan et al., 1993 [40] provided support for the transport of CdMT from the liver to the kidney by showing a gradual uptake of Cd in the kidneys after transplantation of Cd-containing livers to non-Cd exposed rats. Liu et al., 1996 [41] and Liu and Klaassen 1996 [42] showed differences in Cd kinetics between transgenic (MTnull) and wild-type mice. In MTnull mice, elimination of Cd was much faster than in wild-type mice. Cd concentrations in the kidney increased with time in wild-type mice, but not in MTnull mice. These observations support a role of MT in tissue retention and transport of cadmium to the kidney. A review of the presently available evidence, giving general support to the explanatory scheme described in Figure 2, but including detailed and up-to-date information on biochemical pathways explaining Cd kinetics and toxicodynamics was presented by Sabiolic et al. [43]—the reader is referred to this review for further details.

As a result of MT binding and the mentioned time-related distribution changes, adult humans with long-term, low-level exposures, e.g., background exposures in Sweden, have 50 percent of their Cd body burden in the kidneys. In the kidney, the highest Cd concentration is in the kidney cortex (review [3])

### 3.4. Cadmium Excretion—Biological Half-Life

Cd induces MT synthesis in the liver, kidney and other tissues (Section 1 and Section 2) and a large proportion of tissue Cd is bound to MT and becomes trapped in the tissues in this form. This explains the long biological half-lives of cadmium in tissues of humans and animals. Only 0.01–0.02 percent per day of the Cd body burden is excreted in urine and feces. The biological half-life of Cd in human tissues is very long in the phase of Cd accumulation in the kidney. When the Cd level in the kidney cortex reaches a concentration that causes renal tubular dysfunction (see Section 4.1), urinary Cd excretion increases dramatically and the half-life of Cd in the kidney decreases. 

In the accumulation phase, the half-life in human tissues such as muscles, kidney cortex and the liver is 10–30 years according to estimates based on studies of human tissues and excretion patterns. In blood, there is a fast component (100 days) and a slow component (7–16 years) describing the decreasing levels after cessation of occupational exposure in humans. In animals, the half-life in blood plasma changes from minutes immediately after exposure to days at later observation times (reviewed in [3]). Akerstrom et al., 2013 [44] reported a half-life in the human kidney cortex of 23 years at a kidney cortex Cd concentration of 8 mg/kg and 43 years at 23 mg/kg. The longer retention probably is related to more efficient induction of the synthesis of MT at the somewhat higher Cd levels. Cd concentrations in the kidneys of older humans decrease after age 60, possibly because of the less efficient MT induction in older age groups. 

Urinary Cd excretion takes place by transfer of Cd from the renal tubules into urine and by excretion of a small proportion of glomerular filtrate that is not taken up into renal tubular cells as indicated by animal experiments (reviewed in [3] and Section 3.2). In the accumulation phase, before renal tubular damage is induced, urinary Cd is a good indicator of kidney and body burden of Cd. When a toxic cadmium level is reached in the kidney tubules, tubular reabsorption will be impaired and urinary excretion of Cd will increase dramatically. The relationship between kidney Cd and urine Cd changes when tubular dysfunction is induced. Urinary Cd is to a considerable extent MT-bound both in the accumulation phase and when there is tubular dysfunction [31,45,46,47] see also Section 4.2).

### 3.5. Toxicokinetic Model of Cadmium Accumulation in Kidneys

Although a discussion is ongoing whether adverse effects of Cd on the kidneys or the skeleton should be considered as critical effects, i.e., effects that occur at the lowest external exposures, effects on the kidneys occurs at low exposures and are still considered the critical effect [19]. Kjellstrom and Nordberg 1978 [48] presented a toxicokinetic, multicompartment model for Cd kinetics and accumulation in the kidneys based on the identification of a crucial role of MT, largely as described in Figure 2 (see also [49]). Choudhury et al., 2001 [50] developed this model further, using later evidence. The amended model in combination with calculations based on the distribution of the critical concentration of Cd in the kidney cortex has been successfully used in risk assessments by Diamond et al., 2003 [51], ATSDR 2012 [52] and the International Union on Pure and Applied Chemistry 2018 [19]. In the latter document, these model calculations provided a perspective on findings in epidemiological studies. The calculations show that the lowest exposure level of Cd giving rise to kidney dysfunction is very low.

## 4. Cadmium Toxicity and Its Modulation by Metallothioneins

As mentioned in the introduction, clinical doctors first observed toxic effects of Cd compounds. In 1858, Sovet reported [1] that respiratory and gastrointestinal symptoms occurred in persons using a Cd containing polishing agent. Later publications describe toxic effects in the kidneys, liver and the skeleton as well as reproductive toxicity and cancer in humans and animals (reviewed in [3]). Cd binds to MTs in all tissues (Section 2) and adverse effects in lungs, the skeleton, liver and kidneys as well as cancer [3,19] are likely to be modified by binding to metallothioneins. However, studies of such protective effects in other tissues than the liver and kidneys are few.

### 4.1. Liver Toxicity of Cadmium—Role of Metallothioneins

Piscator 1964 [5] showed that Cd was bound to MT in the liver of animals repeatedly exposed to small doses of Cd. He suggested a protective role for MT for tissue toxicity. As mentioned in Section 3.3, Cd salts given to experimental animals in a single large dose goes primarily to the liver where it causes damage. Wisniewska-Knypl and Jablonska 1970 [53] and Nordberg et al., 1971 [8] showed that Cd was bound to MT in the liver of Cd exposed animals and suggested that binding would provide protection against toxicity. After a single large dose of Cd there was liver toxicity and MT binding in liver tissue did not occur until several days after dosing [8]. When animals were pretreated with small doses of cadmium and then given a large dose, there was no liver toxicity and Cd was bound to MT [8]. Goering and Klaassen 1984 [54] showed that resistance to hepatoxicity was due to presynthesized MT. These early studies of a role of MT in protecting the liver have later been followed up and expanded with detailed biochemical and morphological investigations, reviewed by Sabolic et al., 2010 [43]. However, while liver toxicity occurs in experimental animals given comparatively large doses of Cd it is not often found in humans, because most exposures are to lower doses for longer periods of time. The following section focuses on kidney toxicity, since the kidney effects have long been and still are considered critical effects in long-term Cd exposures [19].

### 4.2. Kidney Toxicity of Cadmium—Role of Metallothioneins and Their Cd/Zn Content

After a single high dose of Cd, toxic effects appear in reproductive organs and the liver. One or a few days after a single dose in animals, an increased proportion of liver Cd is bound to metallothioneins, mainly MT-1 and MT-2 [8,55]. As mentioned in Section 3.3, MT-bound Cd is released from the liver into blood plasma and subsequently filtered through the glomerular membrane in the kidneys. From the glomerular filtrate Cd-MT is taken up by the proximal renal tubules (Figure 2). In the tubular cells, CdMT enters the lysosomes [56] where Cd ions are released from MT and reaches other subcellular organelles Later findings reviewed by Sabolic 2010 [43]. Released Cd ions cause renal tubular injury if protective MT is not available to bind the released Cd. An injected bolus dose of CdMT in animals with no prior cadmium exposure will cause renal tubular damage at a concentration in the whole kidney of 9 ug/g wet wt. corresponding to kidney cortex level of 11 ug/g as shown 1975 [57]. Much higher tissue levels are required to induce renal tubular damage in animals with long term exposure to cadmium, for example, in the studies by Nordberg et al., 1971 [8] and Nordberg and Piscator 1972 [58], tubular toxicity in the kidney did not appear until the concentration in the whole kidney reached 130–170 µg/g. Nordberg et al., 1975 [57], suggested an explanation for the different tissue levels related to toxicity. After a single injection of CdMT, the fast transport and uptake of CdMT in kidney tubules causes intracellular release of considerable amounts of Cd-ions, because they are released from MT when the protein is degraded. Such release causes renal tubular toxicity at relatively low Cd levels in total tissue. These levels (ca 10 µg/g tissue) would be minimum levels in kidney cortex for tubular injury from Cd. In long term exposures; higher levels of total tissue Cd are tolerated because there is time for local MT synthesis to take place, sequestering Cd ions. In long-term exposures, tubular kidney injury occurs at even higher tissue levels when the maximum level of local synthesis of MT is reached. Then, the sequestering action of MT will be insufficient. Nordberg et al., 1994 [59] also showed the protective effect of tissue MT in kidneys, sequestering Cd from sensitive membrane binding sites in the kidney tubules of animals injected with CdMT. Other scientists subsequently confirmed these findings [41,42] in MT null mice. In such mice Cd accumulation in the kidney is limited and because there is no MT-protection of renal tissue, renal damage occurs at low tissue levels (see also [60]).

**Figure 3:**
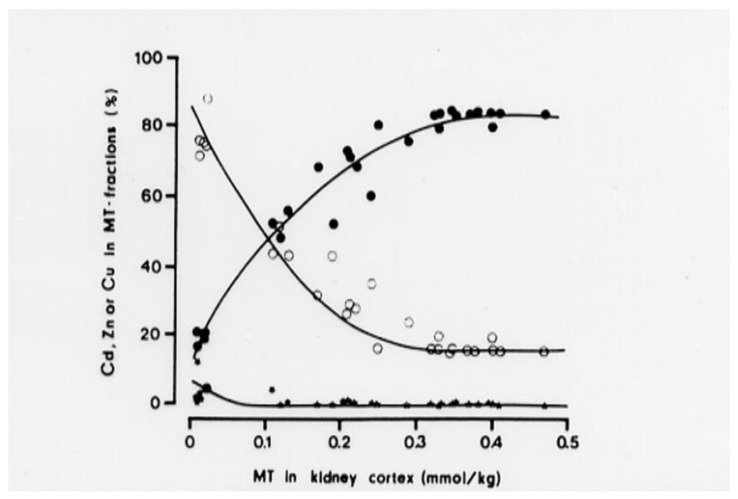
The relative concentrations (percent) of Cd (filled circles), Zn (open circles) and copper (black dots) in MT fractions in relation to the total MT concentration. MT isolated from the kidneys of rabbits with varying exposure to Cd [61].

Studies by Elinder et al. (1987) [61] of Cd-exposed animals (with intact MT synthesis) demonstrated that in MT fractions (MT-1 and MT-2) isolated from the kidneys by gel-chromatography, the molar Cd/Zn quotient increases with increasing level of MT in kidney tissue (Figure 3). The MT-level is proportional to the Cd tissue level and the cumulative exposure to Cd. The change in Cd/Zn quotient occurs because Cd binds stronger to SH-groups in MT than Zn. Cd thus replaces Zn in the protein. When the Cd/Zn quotient in MT is low, cells are offered protection from Cd-toxicity. When the Cd/Zn quotient increases, there are fewer Zn- sites available for Cd to interact with and protection is less efficient. At 0.1 mmol MT, i.e., 0.5 mmol Cd (55 mg Cd/kg) in kidney cortex, protection is impaired and susceptibility to tubular dysfunction increases. When the Cd/Zn quotient is 6, i.e., 6 of the 7 metal binding sites in MT are occupied by Cd, there was renal dysfunction in all animals (at 0.4–0.5 mmol MT Figure 3). MT with almost all binding sites occupied by Cd, cannot bind more Cd and the sequestering function of MT is exhausted. The findings show how MT acts in cellular protection against Cd. These findings explain why there is protection up to a specific critical concentration in renal tubular cells. When total cellular Cd concentrations increase above this level toxic effects occur because of Cd interference with Zn-dependent enzymes and membrane functions [62,63]. These findings support the model (Figure 2) that CdMT, after uptake into renal tubular cells and transfer to lysosomes, releases Cd that interferes with cellular function [56,59,64]. Some evidence [65] suggests a role for zinc transporter ZIP8 for expression of renal toxicity of Cd. Reactive oxygen species are formed when Cd exerts its toxic effects on renal tubular cells [66], but we do not know exactly how different events influence the outcome. However, there is evidence in humans exposed to Cd for long time periods that tubular proteinuria develops when the Cd concentration in the kidney cortex exceeds 80–200 mg/kg [3,19,67]. Although it should be possible to use information about biochemical pathways to refine quantitative risk assessments, such refined models are not yet available. The explanatory model in Figure 2 remains valid even if it does not describe the detailed molecular pathways recently discovered (see above and reviews [11,43]). In addition, Zn status is important because it probably influences the likelihood of Cd replacing Zn in MT and the likelihood of Cd interfering with sensitive molecular intracellular targets. The importance of Zn status was documented in humans [68] residing in a cadmium contaminated area of China. At comparable Cd exposures, persons with a good zinc status had a considerably lower prevalence of renal tubular dysfunction compared to those with low serum and hair Zn.

### 4.3. Studies in Humans on Cadmium and Metallothionein

#### 4.3.1. Urinary Metallothionein as a Biomarker of Kidney Dysfunction

As mentioned in Section 3.4 a large proportion of Cd in urine is bound to MT [31,45,69]. Because dysfunction of kidney tubules means a deficient reabsorption of all low-molecular-weight proteins (including MT) from primary urine, there will be increased excretion of these proteins in urine. The authors who showed that Cd is bound to MT in urine also showed that Cd-exposed persons with tubular dysfunction excrete more MT in urine than non-Cd exposed persons. In an epidemiological study, Shaikh et al., 1990 [70] included 3168 men and women in a cadmium-polluted area of Japan and found increased metallothionein excretion among those with Cd-induced renal tubular dysfunction. Increased urinary MT excretion in relation to occurrence of Cd-related renal dysfunction was also found in cadmium-exposed workers [46] and in persons with type 2 diabetes [47].

#### 4.3.2. Metallothionein Gene Expression in Peripheral Lymphocytes—A Biomarker of Tissue Susceptibility to Cadmium Toxicity

The foregoing text reviewed evidence showing that, MT serves as an efficient intracellular scavenger for cadmium decreasing its toxicity by binding Cd in a number of tissues (reviews [60,71]). Studies in Cd-exposed animals and humans show Cd induced MT synthesis in liver and kidneys. By in vitro Cd exposure of peripheral blood lymphocytes (PBLs), the inducibility of MT and MTmRNA was measured by RT PCR (i.e., MT gene expression (MT-GE) [72]. Lu et al. [72] performed such measurements and examined their possible use as a biomarker of general tissue detoxification by MT. They recruited Cd exposed workers in Guangxi province, China. In addition to the measurements of MT-GE, the studies of the works included urinary Cd as biomarker of exposure and NAG in Urine (UNAG) as effect biomarker. The results showed an increased level of urinary NAG (UNAG) in relation to increased levels of Cd in urine. Workers with high levels of MTmRNA in PBLs had lower NAG levels in urine than those with low MTmRNA levels in PBLs [72], when compared at similar levels of UCd. These results support the hypothesis that the induced level of MTmRNA in PBLs reflect MT expression level both in PBLs and in kidney cortex. MT-GE in PBLs, thus can be used as a biomarker of tissue susceptibility to cadmium toxicity. 

Another study included a group from the general population [73]. Studies of farmers in a Cd polluted area in China included measurements of MT-GE in PBLs at comparable levels of UCd, those with high MT-GE excreted less urinary NAG than those with low MT-GE (difference statistically significant at UCd > 10µg/g Crea *p* < 0.001).

The mentioned studies in Cd-exposed workers and farmers show that MT-GE in PBLs can be used as a biomarker of susceptibility to Cd toxicity. However, the in vitro Cd treatment of fresh PBLs is a demanding requirement in field studies. There is a need to develop methods more suitable for field studies and these methods should be standardized in order for results to be comparable among laboratories [74]. Although occupationally exposed groups have been studied [75] large population studies are not yet available.

#### 4.3.3. Autoantibodies against MT in Blood Plasma—A Biomarker of Susceptibility to Cadmium Nephrotoxicity

Jin et al. [76] found a high frequency of elevated levels of antibodies against MT in sera of patients with metal allergy. This finding stimulated our interest in the possibility that such antibodies might interfere with MT protection in tissues of animals and humans. This was the general background for the following studies: Chen et al., 2006 [46] measured autoantibodies against MT (MTab) in blood plasma by ELISA. In smelter workers and controls in Hunan province, China we measured MTab in blood plasma, UCd, UNAG and UB2M (urinary beta-2-microglobulin). There were increased levels of MTab in relation to increased UNAG or UB2M. At comparable levels of UCd, there was an odds ratio of 4.2 (CI 1.2–14) for tubular dysfunction for persons with increased levels of MTab relative to those with low levels of MTab.

Animal experiments and epidemiological studies provide evidence that diabetes gives rise to an increased sensitivity to development of Cd related kidney dysfunction [77,78] reviewed in [3]. Chen et al. [47] performed a study on the role of MTab for development of renal tubular dysfunction among diabetics in Shanghai, China, with type 2 diabetes. Measurements included MTab in blood plasma, UCd, UNAG, UALB (urinary albumin), UB2M and a number of background variables. UCd was 0.05–4.17, GM 0.38 ug/g crea. There were statistically significant increases of UNAG and UB2M in relation to UCd and a statistically significantly higher odds ratio for tubular dysfunction among those with high MTab versus low MTab. 

In summary, the studies demonstrated that among workers and diabetics, elevated levels of MTab were associated with a higher prevalence of tubular kidney dysfunction compared to those with lower MTab levels. MTab in blood plasma thus is a biomarker of susceptibility to development of Cd-related tubular dysfunction. We do not know the detailed mechanism behind this effect, but it is likely to reflect an interference with tissue protection by MT. 

## 5. Concluding Remarks

Adverse health effects of Cd in humans were first reported 160 years ago and the low molecular weight Cd binding protein metallothionein (MT) was discovered more than 60 years ago. The present review summarized available evidence on the role of MTs in cadmium toxicology as applied to risk assessment. It focused on our own findings from the 1970s and onwards and gave comments on other findings in relation to relevance and applicability in risk assessment. Evidence was presented supporting the scheme (Figure 2) explaining Cd kinetics and interactions with renal targets when eliciting tubular damage in kidneys. Biomarkers like MT gene expression in peripheral blood lymphocytes and MT-antibodies in blood plasma were developed in the last twenty years (Section 4) to be used in refinement of epidemiological studies and to assist in risk assessments. However, to our knowledge the use of these biomarkers at present is very limited. We note that our explanatory scheme for cadmium toxicokinetics and toxicodynamics in the kidneys (Figure 2) is still generally accepted and this toxicokinetic and toxicodynamic model is successfully used for quantitative calculations of risks of renal dysfunction in relation to Cd exposure. Such calculations provide a valuable perspective on findings in epidemiological studies (Section 3.5). Opportunities to use more of the available evidence in risk assessments seems to exist, for example, to use the MT-related biomarkers and to take into account the influence of Zn status (Section 4.1). Even without such refinements of risk assessments, it is obvious that very low exposure levels of Cd give rise to adverse effects on the kidneys and other organs in humans. Partly based on the evidence summarized in the present review, it is recognized that present occupational limit values for Cd in high- and middle-income countries are often higher than desired. Actions for lower values are underway for example in the EU. In some low- and lower–middle-income countries with ongoing artisanal small-scale mining, there are excessive exposures to Cd and other metals and there is an urgent need for improved conditions. This is very important at present when there is growing global demand for metals to combat climate change. We hope that application of the knowledge summarized in this review will assist in improving risk assessments and conditions for population groups exposed to cadmium in various countries.

## Data Availability

Not applicable.
